# Human-capital architecture for biosafety and responsible synthetic biology

**DOI:** 10.3389/fbioe.2026.1946816

**Published:** 2026-10-28

**Authors:** Ibrokhim Y. Abdurakhmonov

**Affiliations:** Ministry of Agriculture of the Republic of Uzbekistan, Center of Genomics and Bioinformatics, Academy of Sciences of Uzbekistan, Tashkent, Uzbekistan

**Keywords:** agricultural biotechnology, biosafety, biosecurity, biotechnology governance, HECI, responsible innovation, synthetic biology, UNESCO

## Abstract

Synthetic biology is entering practical use across medicine, agriculture, industrial biomanufacturing, food systems and environmental applications. This transition creates biosafety and biosecurity challenges that technical safeguards or compliance rules alone cannot solve. The recent UNESCO International Bioethics Committee (IBC) report provides an important ethical perimeter for synthetic biology, but principles must be translated into research habits, institutional cultures and deployment practices. This Perspective proposes a dual-framework model: the UNESCO IBC report defines boundary conditions for responsible biological innovation, while the Humanity, Ethics, Creativity and Imagination (HECI) framework strengthens the human capacities needed to work responsibly within them. Drawing on agricultural education and policy reform in Uzbekistan as a proof-of-principle, the article argues that biosafety in synthetic biology requires not only safer rules, but also scientists, institutions and regulators capable of empathy, judgment, creativity and foresight. The model is intended to stimulate practical experiments in ethics-integrated curricula, design reviews, field-deployment checklists and locally adaptable governance.

## Introduction

Synthetic biology has moved from speculative promise to an operational platform for engineering living systems. It now shapes clinical medicine, agricultural biotechnology, industrial biomanufacturing, food systems, materials science and environmental management. CRISPR-based therapeutics, mRNA platforms, AI-assisted protein design, engineered microbes, cell-free biosystems and automated design-build-test-learn workflows have changed both the speed and the social reach of biological innovation (Endy, 2005; Cohn et al., 2025; Robinson and Nadal, 2025). The question is no longer whether synthetic biology will matter. It is how societies should guide the direction, distribution and trustworthiness of biological design as it moves from laboratories into clinics, farms, factories and ecosystems.

This transition is intensified by convergence. Synthetic biology increasingly interacts with artificial intelligence, robotics, large genomic databases, cloud laboratories, digital supply chains and biofoundry capacity to execute design-build-test-learn (DBTL) biological cycles. These systems increase scientific power, but they also widen the gap between technical capability and institutional preparedness. Laboratories may design faster than regulators can deliberate. Companies may scale faster than publics can understand or contest new applications. Researchers may develop biological functions faster than ethical cultures can adapt to the responsibilities that capacity entails.

These developments create a biosafety and biosecurity challenge, not only a technical one. Biosafety is often associated with containment, risk assessment and prevention of harm, while biosecurity is associated with misuse, deliberate harm and control of dangerous capabilities. Both remain essential. However, synthetic biology also requires better policy and decision-making across the full life cycle of biological design, from sequence access and strain construction to containment, release, monitoring and public accountability (Raybould, 2021). The problem is therefore not whether science should slow down, but whether institutions can build frameworks that keep fast-moving biological innovation scientifically robust, ethically legitimate and publicly useful.

This delay is often discussed as a pacing problem: technical development can outstrip legal, ethical and institutional oversight. For synthetic biology, digital design, distributed platforms and field deployment intensify the problem, moving applications through research, markets and ecosystems faster than formal regulation or public deliberation. This makes anticipatory and adaptive governance a biosafety requirement, not only a policy preference (Marchant, 2011; OECD, 2026).

Agricultural and environmental biotechnology make this challenge particularly visible. Synthetic biology can contribute to crops better adapted to heat, salinity and drought; biological fertilizers and biostimulants that reduce chemical inputs; engineered microbes that support soil health; improved animal vaccines and diagnostics; food traceability; and bio-based materials (Rodrigues et al., 2023). These applications matter to regions already experiencing climate stress, land degradation, water scarcity and food-system vulnerability (Rockström et al., 2009). However, field deployment reveals the limits of a purely technical model. Biological interventions enter ecosystems, supply chains, rural livelihoods, cultural settings and unequal political economies. A design that is elegant in the laboratory may still fail if it is ecologically fragile, socially illegible or institutionally unsupported.

Recent gene-editing risk-governance work reinforces this point. Agricultural applications may raise questions about target and off-target effects, persistence or spread, non-target interactions, food and feed implications, and ecosystem-specific exposure pathways. These concerns do not argue against innovation; they show why deployment should be staged, monitored and assessed in the specific ecological, agricultural and institutional contexts in which engineered biological systems are expected to operate (Agapito-Tenfen et al., 2018; Kawall et al., 2020).

## The UNESCO IBC report and the implementation gap

The UNESCO International Bioethics Committee (IBC) report is one of the most important recent efforts to frame synthetic-biology governance in explicitly ethical terms. It identifies substantive principles, including beneficence and non-maleficence, precaution, protection of future generations, justice and equity, solidarity and planetary sustainability, and links them to procedural values of transparency, accountability and inclusion (UNESCO, 2025; Kurtoğlu et al., 2024). This shifts the field from the narrow question of what can be built to the harder questions of what should be built, for whom, under what risks and with what forms of accountability.

That shift matters because synthetic biology creates uncertainties that cannot be managed as engineering parameters alone. Some concerns are familiar: dual-use risks, accidental release, unequal access, opaque commercial control and the possibility that benefits will accrue to powerful actors while costs are displaced onto less powerful communities. Other concerns are emerging quickly: cyberbiosecurity vulnerabilities in digitally connected laboratories and sequence systems, compression of design cycles by AI tools and experimental directions, including speculative but high-consequence risks such as mirror-life research, where uncertainty may be profound enough to justify strong precaution (Reed and Dunaway, 2019; Richardson et al., 2019; Adamala et al., 2024). In this context, the UNESCO IBC report performs an essential function. It names red lines, argues for precaution where uncertainty is deep and insists that governance should include affected publics rather than only technical experts.

The report’s deeper value lies in distinguishing technical sophistication from moral adequacy. A biological intervention can be scientifically ingenious yet ethically deficient if it externalizes risk, deepens inequality, marginalizes local knowledge or undermines ecological integrity. This corrective matters in a field where novelty can too easily be treated as a proxy for social value.

Although important and timely, the UNESCO IBC report is a governance document and it also has certain limitations. It can define principles, establish expectations and recommend oversight. It cannot, by itself, cultivate the dispositions needed to make those commitments real rather than procedural. It cannot ensure that consultation is sincere rather than symbolic. It cannot teach a synthetic biologist to treat an ethical boundary as a design constraint rather than an obstacle. It cannot generate empathy for farming communities, patients or ecosystems, nor can it substitute for institutional cultures in which researchers internalize responsibility rather than merely satisfy compliance (Nyrup, 2021; Parker, 2026). Governance can draw the perimeter; it cannot alone produce the human judgment required to act wisely within it.

### HECI as a human-capital architecture for biosafety

The Humanity, Ethics, Creativity and Imagination framework, or HECI, offers one way to describe the second layer that responsible synthetic biology now needs. Leonhard (2022), writing as a futurist and technology commentator, originally framed HECI as a necessary complement to science, technology, engineering and mathematics education for preparing people to work responsibly in a technology-intensive future. In Uzbekistan, this idea was subsequently operationalized as a humanistic complement to STEM in agricultural education and innovation policy (Abdurakhmonov et al., 2025; 2026). Although HECI’s original context was education and future-oriented human-capital formation rather than synthetic biology alone, the framework is relevant to the broader challenge of shaping how scientists, institutions and policymakers use powerful technologies under conditions of uncertainty, urgency and public consequence.

That wider origin is useful for biosafety and biosecurity. HECI does not ask scientists to abandon technical ambition. It asks that ambition be formed by four capacities. Humanity emphasizes empathy, dignity, care and responsibility toward communities, ecosystems and future generations. In biological innovation, this means treating patients, farmers, animals, soil, water and biodiversity not merely as variables in a performance system, but as living contexts affected by design decisions. Ethics provides the language of responsibility, fairness and reflection. In HECI, however, ethics is not limited to rule-following. It includes the cultivated ability to ask difficult questions about distributive justice, unintended consequences and legitimate boundaries of intervention (Sandler, 2012; Thompson, 2015).

Creativity is often celebrated in biotechnology, but responsibility rarely disciplines it. In HECI, creativity is the capacity to find context-sensitive solutions within ethical limits. This matters because biosafety governance should not produce paralysis. If precaution is interpreted only as restraint, innovation becomes brittle. Responsible creativity allows scientists and institutions to redesign methods, containment strategies, deployment pathways, monitoring protocols and stakeholder relationships in ways that reduce harm while preserving beneficial possibilities (Buchanan, 1992; Dorst, 2011). Imagination extends this work across time. It enables scenario thinking, anticipation and the ability to look beyond immediate performance metrics toward long-term ecological, social and institutional futures (Stilgoe et al., 2013).

HECI also clarifies what it adds to established responsible-innovation approaches. The anticipation, inclusion, reflexivity and responsiveness (AIRR) framework describes essential procedural dimensions of responsible research and innovation (Stilgoe et al., 2013). While AIRR describes what governance processes should do, HECI addresses the formation problem underneath those processes: who scientists, educators and regulators must become, and what cognitive and affective capacities they must cultivate, for anticipation, inclusion, reflexivity and responsiveness to become everyday scientific practice rather than a checklist. Put differently, AIRR defines important procedural values for responsible innovation, whereas HECI asks how people and institutions can be formed to enact those values in everyday research, biosafety and deployment decisions.

A simple metaphor clarifies the relationship. Synthetic biology provides the biological hardware; STEM supplies the engineering capability; the UNESCO IBC report defines the ethical safety code; and HECI functions as the values-based operating system that shapes how biological power is used. The metaphor is not meant to mechanize ethics. It highlights that powerful biological tools require external safeguards and an internal culture of judgment. Rules can define acceptable boundaries, but people and institutions decide how those boundaries are interpreted in practice. HECI is therefore not a substitute for formal oversight. It complements formal oversight by helping convert an ethical perimeter into scientific culture, educational practice and institutional habit (Figure 1).

**FIGURE 1:**
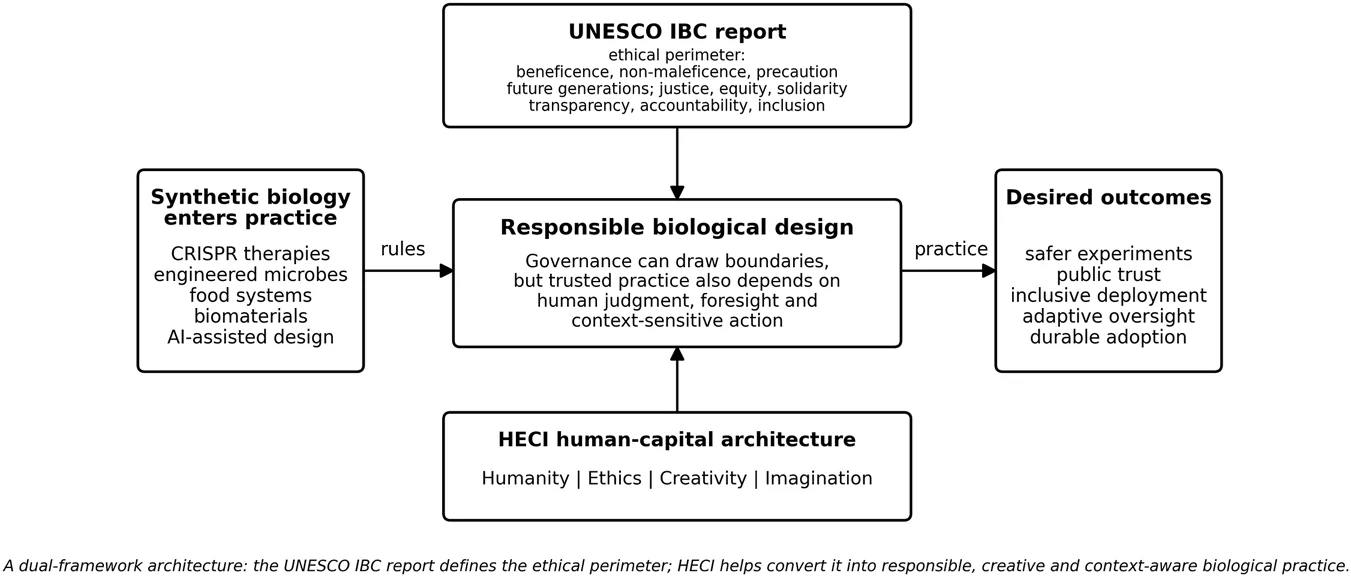
From ethical perimeter to responsible biological practice. Synthetic biology is moving into practical use across medicine, agriculture, food systems, industrial biomanufacturing and environmental applications. The UNESCO IBC report provides an ethical perimeter: principles and procedural expectations that define what responsible synthetic biology must respect. HECI provides a human-capital architecture: Humanity, Ethics, Creativity and Imagination, which helps scientists, institutions and policymakers convert ethical boundaries into trustworthy, context-aware biological design and deployment.

### Uzbekistan as proof-of-principle

A recurring weakness in biotechnology-governance writing is abstraction. Ethical frameworks can be compelling at the level of principle but detached from the settings in which science is taught, regulated and deployed (Feenberg, 2002). Uzbekistan is therefore presented here not as a universal template, but as a proof-of-principle. It shows how a humanistic framework can be embedded within an applied agricultural reform process and used to connect education, innovation policy and public responsibility.

Beginning in 2022, HECI was introduced as part of educational and policy reform in Uzbekistan’s agricultural system. It was developed into a methodological guide, piloted in university instruction (including a 24-h elective course on Humanity, Ethics, Creativity and Imagination approaches), and used to frame discussions on agricultural innovation, sustainability, and science-society relations (Abdurakhmonov et al., 2025; Abdurakhmonov et al., 2026). The context matters. Uzbekistan’s agriculture is economically central, environmentally constrained and politically important. Water scarcity, salinity, climate stress, land-use pressures and the need for diversified rural development make innovation governance unusually consequential. In such a setting, powerful biological or agricultural technologies cannot be evaluated solely by laboratory performance. They must also be judged by farmer trust, institutional capacity, ecological durability and social legitimacy.

At this stage, the Uzbekistan example should be read as evidence of implementation and institutional translation rather than as a quantified efficacy claim. Formal evaluation of HECI’s educational and governance effects remains an important next step; appropriate measures could include pre- and post-course assessment of ethical reasoning, reflective assignments, interviews with students and instructors, and comparison with conventional ethics-add-on instruction.

Concrete examples make the proof-of-principle visible. In wheat production, HECI helped reframe seed vernalization not as an obsolete practice to be dismissed, but as biological wisdom that can be reinterpreted through modern agronomy. In irrigation modernization, it highlighted that concrete-lined canals may save water but should also be designed with attention to trees and vegetation along canal edges; small adaptive measures can prevent a technically efficient intervention from becoming ecologically insensitive. In cotton systems, biochar from cotton stalks illustrates how agricultural residues can be reimagined as circular-economy resources for soil health and carbon management. Future refuge planning for Bt-type or other insect-resistant crops is a biosafety and stewardship example in the same logic. Refuges are not only technical details of crop deployment; they are resistance-management systems that depend on farmer behavior, monitoring, incentives and regulatory design. When HECI is applied to such decisions, the question becomes not only whether the trait performs in one season, but also whether deployment preserves susceptibility in pest populations, reduces future risk for neighboring farmers, and preserves the technology for future generations.

These examples are modest in themselves, but together they show how a human-capital framework can turn ethical principles into habits of care, foresight and redesign. The lesson for synthetic biology is direct. If a country can use HECI to reshape how agricultural students, researchers and policymakers think about irrigation, residue use, crop deployment and public responsibility, then similar formation can be applied to engineered microbes, gene-edited crops, cell-free biomanufacturing, biological containment and environmental biotechnology. The relevant question is not whether humanistic formation is as precise as molecular engineering. It is whether molecular engineering can remain trustworthy without it.

The Global South dimension is also important. Synthetic-biology governance remains strongly shaped by North American and Western European policy literature. Yet many consequential applications of synthetic biology, particularly in food security, climate adaptation, low-input agriculture, animal health, water management and distributed bioeconomies, will unfold in regions with different development priorities, institutional histories and social risks. A perspective from Central Asian agricultural reform, therefore, broadens rather than narrows the discussion of responsible innovation.

## Discussion: toward experimental cultures of responsibility

The practical implication is that biosafety and biosecurity should be treated as part of good biological research design, not as external compliance burdens. In a DBTL cycle, responsible innovation should shape each stage. Design should include early reflection on beneficiaries, ecological contexts and plausible misuse. Build should include containment, traceability and governance of biological parts, strains, data and protocols. The test should include not only performance metrics but also failure modes, reversibility, environmental monitoring and the distribution of risk. Learning should include feedback from users, communities, regulators and ecosystems, not only from assays and models.

This reframing matters most for applications that leave controlled laboratory settings. Engineered soil microbes, gene-edited crops, biological fertilizers, gene drives, synthetic microbiomes and environmental remediation systems require more than technical validation. They require staged deployment, adaptive monitoring, meaningful public engagement and institutional capacity to respond when biological systems behave differently in field conditions than they did in models. These are not soft additions to science. They help make biological conclusions more robust under real-world conditions.

Universities and research institutes therefore need to train synthetic biologists not only as engineers of living systems but also as stewards of powerful technologies. A brief ethics module at the end of a technical curriculum is unlikely to change research culture. Integrated formation is more durable. HECI offers one vocabulary for this integration. Other frameworks may do this differently, but formation is not optional if synthetic biology is to retain trust as it scales.

Regulators and governments face a parallel challenge. Oversight remains essential, particularly for biosafety, biosecurity, environmental release and dual-use concerns. However, regulation that only prohibits harm may produce formal compliance without responsible internalization. Capability-building is therefore a governance function. It includes investment in ethical literacy, participatory institutions, public communication, interdisciplinary review and post-deployment monitoring. The aim is not to weaken regulation, but to ensure people and institutions can inhabit it and use it wisely. The same logic applies to industry, funders and international organizations (Table 1).

**TABLE 1:** Practical questions for a dual-framework approach to biosafety and responsible synthetic-biology governance.

Actor	Question for practice	Operational implication
Synthetic biologists	Have we designed this project only for performance, or also for safety, reversibility, monitoring and legitimacy?	The UNESCO IBC report frames duties of safety and accountability; HECI adds judgment, empathy, creativity and foresight
Universities and institutes	Are we training synthetic biologists only as engineers, or also as stewards of powerful biological systems?	The UNESCO IBC report defines the expectation; HECI helps operationalize it in curricula and institutional habits
Regulators and governments	Do our rules prohibit harm while also building capacity to innovate responsibly?	The UNESCO IBC report provides the ethical perimeter; HECI supports educational and institutional implementation
Industry and funders	Are speed and scale balanced with transparency, inclusion and distributive fairness?	The UNESCO IBC report identifies justice and accountability; HECI strengthens trust-building and context-sensitive design
International organizations	Are governance models globally coherent yet locally adaptable?	The UNESCO IBC report offers shared ethical language; HECI helps translate that language into local practice

Implementation must also reach actors outside professional synthetic-biology laboratories. Clinicians, extension agents, farmers, citizen users, local authorities, investors and informed publics may influence whether engineered organisms, bioinputs, gene-edited products or biomedical applications are adopted, resisted, misused or monitored. The UNESCO IBC report contributes by making transparency, accountability and inclusion part of governance rather than optional outreach. HECI contributes by translating those procedural expectations into educational and deliberative practices that help non-specialists ask concrete questions about ecological release, biomedical claims, aesthetic interventions, local benefit, reversibility and post-deployment responsibility. In Uzbekistan, the present scale-up pathway has been educational and institutional rather than a dedicated synthetic-biology public campaign: HECI has been introduced through university instruction, methodological guidance and policy discussion in agricultural innovation. The next step is to adapt such formation into biosafety-literacy materials and deliberative forums for clinicians, extension services, farmers, local communities and regulators.

The second challenge is that synthetic biology can narrow as well as expand choices. Platforms may concentrate around species, traits or delivery systems that are technically easier to engineer or commercially attractive, while alternative, locally preferred or lower-risk approaches receive less attention. Unequal access can also mean that some communities bear ecological or market risks without sharing proportionate benefits. Here, the UNESCO IBC report provides normative tests: precaution when ecological change may be difficult to reverse, justice and equity when benefits and burdens are uneven, and planetary sustainability when interventions may displace ecological diversity. HECI translates those tests into design questions: whose problem is being solved, whether non-synthetic or lower-risk options have been considered, whether deployment can be monitored or reversed, whether local users can refuse or adapt the technology, and whether long-term stewardship capacity exists. In this sense, ecological lock-in, technology dominance and unequal access are not external social issues; they are part of responsible biosafety and biosecurity governance.

Operationally, such governance requires horizon scanning, monitoring and assessment. Horizon scanning can identify emerging organisms, platforms and deployment pathways before they become widespread. Monitoring can test whether engineered systems behave as expected after use or release. Assessment can connect observed outcomes back to risk hypotheses, regulatory decisions and institutional learning. These activities should be linked to capacity building, access to technology and knowledge sharing, especially where synthetic-biology applications may arrive before local institutions have sufficient technical capacity (Ballester et al., 2023; Miklau et al., 2024; OECD, 2026).

This argument should be tested, not merely asserted. Synthetic-biology programs could pilot HECI-informed design reviews, compare ethics-integrated and ethics-add-on curricula, develop field-deployment checklists that combine UNESCO IBC principles with human-capital prompts and evaluate whether such approaches improve stakeholder trust, anticipatory capacity and responsible design decisions. These are empirical and institutional questions that biologists, educators and governance scholars can address together. Such pilots could also test whether horizon scanning and post-deployment monitoring improve the translation of ethical principles into practical biosafety decisions.

The broader lesson extends beyond a single framework. Powerful technologies fail society not only when rules are absent, but also when people and institutions lack the habits needed to apply rules under uncertainty. Synthetic biology is an especially vivid case because it intervenes in living systems. Its future will depend not only on better tools, safer parts and stronger oversight, but also on the formation of scientists and institutions capable of using biological power with judgment. Compliance may keep science within bounds. Wisdom determines what science becomes within them.

## Data Availability

No datasets were generated or analyzed in this study. All sources discussed are publicly available and cited in the article.
